# Robotically-Assisted Coronary Artery Bypass Grafting

**DOI:** 10.4061/2010/175450

**Published:** 2010-03-18

**Authors:** Thierry A. Folliguet, Alain Dibie, François Philippe, Fabrice Larrazet, Michel S. Slama, François Laborde

**Affiliations:** Department of Cardio-Vascular Surgery, L'Institut Mutualiste Montsouris, 42 Boulevard Jourdan, 75014 Paris, France

## Abstract

*Objectives*. Robotic surgery enables to perform coronary surgery totally endoscopically. This report describes our experience using the da Vinci system for coronary artery bypass surgery. *Methods*. Patients requiring single-or-double vessel revascularization were eligible. The procedure was performed without cardiopulmonary bypass on a beating heart. *Results*. From April 2004 to May 2008, fifty-six patients were enrolled in the study. Twenty-four patients underwent robotic harvesting of the mammary conduit followed by minimal invasive direct coronary artery bypass (MIDCAB), and twenty-three patients had a totally endoscopic coronary artery bypass (TECAB) grafting. Nine patients (16%) were converted to open techniques. The mean total operating time for TECAB was 372 ± 104 minutes and for MIDCAB was 220 ± 69 minutes. Followup was complete for all patients up to one year. There was one hospital death following MIDCAB and two deaths at follow up. Forty-eight patients had an angiogram or CT scan revealing occlusion or anastomotic stenoses (>50%) in 6 patients. Overall permeability was 92%. *Conclusions*. Robotic surgery can be performed with promising results.

## 1. Introduction

Coronary artery bypass grafting (CABG) provides complete revascularization with excellent long-term results and a low mortality. However it generates significant complications and important costs. The tendency is actually to perform operations through smaller and smaller incisions as to reduce hospital stay and to fasten postoperative recovery. More recently robotic-assisted thoracoscopic coronary surgery provides the ability to perform revascularization either totally endoscopic (TECAB) [1–3] or via small thoracostomies (MIDCAB) [4–6]. Our institution initiated robotic cardiac surgery in 2004 using the da Vinci surgical system (Intuitive surgical, Sunnyvale, CA), and we have completed over 150 cases of robotic cardiac surgeries. The current study describes our experience with robotic coronary artery revascularization, specifically addressing feasibility safety and efficacy while discussing its potential value and limitations to the patient. 

## 2. Patients and Methods

Patients with single-or-double vessel coronary artery disease referred for surgical revascularization were eligible for the study. We report our experience from April 2004 to June 2008 which includes 56 patients. All patients gave informed consent for coronary artery bypass surgery using the da Vinci surgical system (first generation).

### 2.1. Training Protocol

Before starting the series we underwent a stepwise training program including basic da Vinci training, robotically-assisted IMA mobilizations, and TECAB specific training.

### 2.2. Inclusion and Exclusion Criteria

All patients considered eligible for enrollment were reviewed by a surgeon and a cardiologist who reviewed all coronary angiograms. Patients who had a single occluded artery or an instent restenosis were considered, as well as patient with double-vessel lesion, or patients who could benefit from a hybrid procedure. Hybrid procedures were defined as patients with two lesions: one which could easily be treated by angioplasty, whereas the other lesion was better suited for surgical revascularization (occluded artery, long calcified type C lesion).

All patients had a preoperative surgical and anesthesiologist visit which included medical history, physical examination, pulmonary function tests, computer tomography of the chest and/or gated coronary CT scan, and echocardiography. Based on the information and the established inclusion-exclusion criteria the patient was offered robotically-assisted coronary revascularization. Exclusion criteria included left ventricular ejection fraction <0.30, emergency surgery, pulmonary edema, inability to ventilate on one lung (asthma, chronic obstructive pulmonary disease, pulmonary fibrosis), hemodynamic instability, anatomy unsuitable for endoscopic surgery (scoliosis, kyphosis, morbid obesity), previous lung surgery, pleural adhesions visible on CT scan, and patient unwilling to sign an informed consent.

Depending on the location of the coronary as well as the caliber and the degree of calcifications, a TECAB or a MIDCAB was chosen. If the circumflex or the right coronary arteries needed to be revascularized, a MIDCAB was chosen, as well as for patients needing a double revascularization left anterior descending (LAD) diagonal. A TECAB was proposed only for patients in need of revascularization of an LAD or a large diagonal branch.

### 2.3. Operative Technique

Anesthesia was slightly modified compared to patients undergoing CABG with ECC. Betablocants are continued in the hospital and given the day of surgery in order to maintain a cardiac rhythm between 50 and 60 beats per minute. Anesthesia was maintained with reduced doses, Sulfentanyl (Sufenta) 2 mcg/kg, Fluritrazepam (Narcozep) 0.03 mg/kg, and Bromure of Pancuronium (Pavulon) 0,1 mg/kg, associated with an inhalation agent (Enflurane, Ethrane). In case of tachycardia heart rate can be slowed with intrasvenous betablocking agents (Esmolol IV), or calcium channel blockers (Diltiazem). All patients were monitored with a radial continuous cardiac output monitor.

Heparin doses were reduced 50 UI/Kg IV, without any protamin reversal.

A double-lumen endotracheal tube is placed during the surgery; it is subsequently changed to a single-lumen tube at the end of the procedure.

A camera was inserted through a 5th intercostal space midaxillary line and two 8 mm robotic instruments trocars were placed in the 2nd and 7th intercostal space midaxillary line in order to obtain a triangulation with the camera. The left or right mammary artery was dissected entirely from its insertion on the subclavian artery to its bifurcation. The pericardium was then open over the target vessel and an endoscopic stabilizer was inserted through a 12 mm port placed in the subxiphoid region and positioned over the target vessel. After heparinization, occlusion of the artery was done with silastic loops; arteriotomy was performed as an end to side anastomosis using running 8-0 monofilament (Goretex or Prolene) sutures. At completion of the anastomosis all trocars are removed and two chest tubes are placed. 

 During mammary harvesting both lungs were ventilated while during coronary artery anastomosis single right lung ventilation was maintained. The procedure has been described in detail in other studies [7, 8].

All TECAB or MIDCAB was performed with the endoscopic stabilizer which was either placed Trans xiphoid or directly through the mini thoracic incision. In fifteen patients with incomplete coronary artery occlusion, we placed a coronary shunt, and the anastomosis was performed over a shunt. No patient needed pump assistance through groin cannulation.

When a double bypass was performed, both mammary arteries were harvested through the left chest. The anastomosis of the right mammary artery to the right coronary was made through a small 4th right intercostals thoracotomy. The endostabilizer was placed directly through the incision and the anastomosis was hand sawn. When the circumflex artery was chosen, the incision was slightly more posterior on the left 4th intercostals space. The endo stabilizer was placed through the incision, and the anastomosis was hand made.

### 2.4. Exclusion versus Conversion

Once the patients were enrolled, the surgical team decided to perform either a MIDCAB or a TECAB. Intraoperative conversions were defined as cases in which a TECAB was scheduled but had to be abandoned for various reasons and converted to either a MIDCAB or a midline sternotomy.

### 2.5. Followup Postoperative Assessment

All immediate postsurgical clinical information was recorded per standard hospital practice and this included a postoperative 12-lead ECG, chest X-ray, cardiac enzymes, and echocardiogram.

Patients had a followup visit between one to three months. Supplemental postoperative studies and physical exam, resting 12-lead ECG, stress ECG or SPECT, and transthoracic echo were obtained. All patients were offered a control angiogram after three months, and in case of refusal, a gated CT scan was performed. All angiograms or CT scan was seen and reviewed by the team which included a surgeon and an angiographer.

All information was prospectively entered in the database.

All patients continued to be followed through regular information obtained by the referring cardiologist.

Comparisons between groups were performed by the standard *X*_2_ test (or Fisher's exact test when an expected frequency was less than 5) to compare categorical variables and operative mortality, and the* t* test to compare continuous variables. Statistical significance was accepted at* P*  <  .05.

## 3. Results

### 3.1. Patient Population

A total of 56 patients were potential candidates for robotic-assisted surgery and agreed to sign an informed consent. Demographics and baseline characteristics are summarized in Table 1. The procedures scheduled were a TECAB in 32 patients and a MIDCAB in 24 patients. The TECAB procedure was proposed if the coronary anatomy seemed favorable (large vessel > 3 mm, no major calcification, no intramyocardial course), and if the ejection fraction was normal. In other cases a MIDCAB was proposed with harvesting of the LIMA or RIMA robotically and the coronary anastomosis was done through a small thoracotomy (left or right depending on the vessel).

Two patients were redo patients, both patients with patent left-sided graft and occluded right venous grafts. Twelve patients underwent hybrid procedures with surgical revascularization on the LAD and angioplasty of another vessel. All patients had a LIMA LAD anastomosis performed while the other vessels stented were the right coronary (8 patients) and the circumflex (4 patients). The order of the procedures varied as some patients had angioplasty prior to surgery (4 patients), or surgery first (8 patients). No procedures were done simultaneously. Patients were always discharged after the first procedure and readmitted for the second procedure.

The primary efficacy endpoint of the study was the composite endpoint of LIMA-LAD graft patency and freedom from target vessel reintervention during the period of observation. The primary safety endpoints of the study were freedom from major adverse cardiac events, including mortality, target vessel reintervention, and myocardial infarction. 

For each case, intraoperative times were determined based on perfusion, anesthesia, and operative records or were explicitly recorded during the operation. The details of the surgical procedure, including revascularization scheme, steps of the surgical procedure that were completed robotically, conversion to alternate techniques, time to conversion, and reason for conversion, were documented in the operative report forms.

### 3.2. In-Hospital Morbidity and In-Hospital Outcome

Nine patients in the TECAB group had to be converted, 3 to sternotomy and 6 to MIDCAB. Table 2 lists the cause of conversion and the outcome of the patients. No patients in the MIDCAB group had to be converted to sternotomy.

### 3.3. Operative and ICU Data

**Figure 1:**
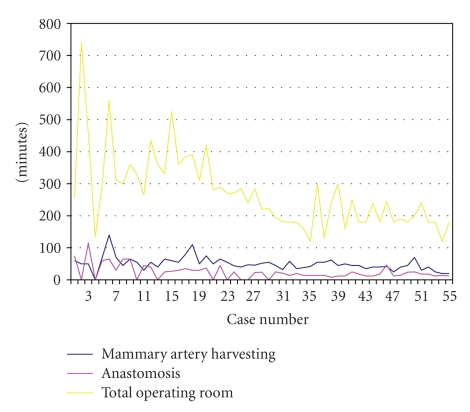
Operative time learning curves.

In the 56 patients a total of 59 anastomosis were completed as 4 patients had a double bypass graft. The LAD was grafted in 77% (42), diagonal branches in 5% (5), marginal branches in 3% (2), and the right coronary artery and posterior descending artery in 3% (3). Total operating time mammary artery harvest and anastomosis time are reported in Table 3. The “learning curve” is depicted graphically in Figure 1. The mean reduction of time with increasing experience was statistically significant for total operative time, but not for anastomosis or mammary artery harvesting.

Postoperative ventilation time was 6 ± 12 hours, ICU length was 52 ± 23 hours, and hospital length of stay was 7.1 ± 3.5 days (range: 5–92 days).

### 3.4. Late Outcome

All patients were followed up for a minimum of one year and the mean follow up was 13 ± 36 months. One patient died in the hospital (1.7%) of respiratory insufficiency at 6 days postoperatively. Autopsy revealed ARDS and a patent LIMA-LAD graft. Two patients died during follow up. One patient died of mesenteric ischemia as revealed by autopsy at 4 months. One patient developed gastric carcinoma and died 9 months post-operatively. Of the 56 patients operated, 48 patients agreed to have a postoperative angiogram (44 patients) or a gated CT scan (4 patients). Eight patients asymptomatic refused to undergo either coronary angiogram or gated scan. The controls were performed at a minimum of three months and a total of 52 anastomosis were controlled, with occlusion of the graft in 4 patients and a significant anastomotic stenosis (<50%) in two patients. Of the 6 patients with anastomosis dysfunction, three patients were reoperated: one underwent angioplasty and two asymptomatic patients were treated medically. Four patients refused either angiography or CT scan but are alive and symptom free one year after surgery. Therefore the permeability rate is 48/52 or 92% and the target vessel reintervention rate is 4/56, or 7%.

All together MACE events occurred in 8/56 patients (14%) and are listed in Table 4: one in-hospital death, one perioperative myocardial infarction, and 4 vessels reinterventions (as described above). Transfusion was required in only 4 patients (7.1%).

## 4. Comment

Single-vessel occluded coronary artery represents a small group of patients with atheromatous coronary disease and most of these patients are treated by mammary vessels revascularization done through a sternotomy with or without extracorporeal circulation according to the surgeon's preference and experience. Other patients present multiple coronary vessel disease with one or two occluded vessels. Some of these patients can be treated by a combination of surgery for the occluded vessels and angioplasty on the other vessels (hybrid revascularization) with excellent results and a low morbidity [9–12]. Another possibility is to perform a MIDCAB with harvesting of the mammary vessel and coronary anastomosis done through a mini incision [13, 14]. Robotic surgery represents another option as the mammary vessels can be harvested and the anastomosis done totally endoscopically (TECAB) [8, 15]. Certainly many reports have shown that TECAB can be done with a permeability rate of 90% [16, 17]. However this permeability rate is certainly inferior to surgery performed with extracorporeal bypass on an arrested heart through a sternotomy, and this is reflected in our studies which shows an 11% failure rate of the anastomosis. Therefore the real question is whether TECAB surgery should be performed or attempted since 5-year results report 87% free from reintervention on the grafted vessel [18]. It is difficult to answer this question as also two possibilities exist: TECAB with ECC or on a beating heart. The real answer in order to improve results of robotic surgery is patient's selection. The robotic system now available allows performing safely one-vessel revascularization and, therefore, should be reserved for this group of patients. Obviously double-vessel or triple vessel revascularization can be done, but with increasing long operating time [19–22]. We have noted in our series that the longer the procedure is, the more pulmonary comorbidities can occur, and therefore the ICU time is increased. Also the anastomosis which is one of the crucial elements of the procedure is always at the end of the procedure when the team is loosing some of its concentration. Therefore when the procedure becomes too complex, we recommend switching to a MIDCAB procedure which allows excellent results without long operating time [22]. The selection of patients is extremely important for this procedure. There are two aspects which should be carefully reviewed. First the patient's comorbidities should be reviewed. As we have already mentioned, all patients with pulmonary disease and/or poor ejection fraction should be excluded since poor hemodynamic or low cardiac output can develop during the procedure due to CO_2_ insufflations which decreases venous return, and also cardiac stabilization which sometimes decreases cardiac output. For these reasons we routinely monitor cardiac output throughout the procedure with a radial monitor. When cardiac output decreases and is not improved despite simple measures (adding extra volume), we generally convert to a MIDCAB procedure [23]. The other aspects which should be carefully reviewed are the location, quality, and trajectory of the target vessel. Therefore, we have performed preoperative gated CT scan which allows us to appreciate the degree of calcifications and the portion of the artery either intramyocardial or epicardial. This remains more important than in open surgery as the artery sometimes can be quite difficult to find if the vessel is in the fat or intramyocardial. Also if the vessel is calcified, it can be more difficult to perform the anastomosis with the robot. So we think that diffuse calcified and intramyocardial vessels should be exclusion criteria for the TECAB procedure. Finally the diameter of the vessel is important. It is difficult to select a number as to which to recommend not to attempt a TECAB, but certainly the bigger the vessel, the easier the anastomosis and better the result will be. Occluded arteries are an ideal situation since there is rarely any ischemia during vessels occlusion, and therefore the time to perform the coronary anastomosis is not an issue. Another interesting group of patients are redo coronary surgery. Sometimes only one vessel needs to be revascularised as the other grafts are still patent and certainly mini access or robotic surgery can be a simple option [24]. It avoids redo sternotomy and its complications; the stabilization of the heart is usually simpler as the adhesions decrease the beating motion of the myocardium. However it can be sometimes difficult to find the target vessel and a preoperative CT scan can help in finding the vessel.

The length of stay in this study was 7 days which is not much different then for sternotomy patients. This is certainly much higher than in various countries but is the norm in our country where patients are kept around a week in the hospital before being sent to a rehabilitation center. However most of the patients (48/56) were discharged directly home, whereas the patient who underwent a sternotomy was sent to a rehabilitation center.

## 5. Conclusion

Robotic coronary surgery is basically feasible and can be performed, but with a high conversion rate and a lower permeability rate than conventional surgery. Selection of the patient and also of the target vessel is extremely important to avoid conversion or poor vessel patency. In case of hemodynamic instability or increased difficulty occurring during the surgery, quick conversion should be performed. New techniques are needed in order to improve permeability rates.

## Figures and Tables

**Table 1:** Patient Demographics.

Variable	Number (%)
Number of patients	56
male	46 (80)
female	10 (20)
Mean age (years)	66 ± 11 (38–82)
Hypertension	32 (57)
Diabetes	14 (25)
History of PTCA restenosis	26 (46 )
Myocardial infarction	37 (66)
Chronic renal failure	2 (3)
Angina	54 (96)
Average preoperative LVEF	49 ± 6 (30–65)
Redo surgery	2 (3)
EuroScore	3.9 ± 2 (0–9)

PTCA: percutaneous angioplasty.

**Table 2:** Conversion and outcome.

Conversion	Cause	Angiogram	Outcome
No 2 Sternotomy	Intramyocardial LAD	Patent	Alive
No 4 Sternotomy	Myocardial injury with trocar	Patent	Alive
No 10 Sternotomy	Calcified LAD Myocardial ischemia	Patent	Died of mesenteric ischemia (3 months)
No 13 MIDCAB	Intramyocardial LAD	Patent	Alive
No 16 MIDCAB	Small calcified LAD	Patent	Alive
No 26 MIDCAB	Robotic arm technical failure	Patent	Alive
No 29 MIDCAB	Epicardial fat inability to visualize LAD	Patent	Alive
No 44 MIDCAB	Ventilation problem	Patent	Alive
No 52 MIDCAB	Robotic instruments technical failure	Patent	Alive

**Table 3:** Operative variable.

Variable	Total *n* = 56
Total OR time (minutes)	274 ± 118
	median 255
Mammary artery harvest (minutes)	53 ± 23
	median 50
Anastomosis	30 ± 23
	median 24
Double bypass	4
LAD diagonal	2
LAD right	2
Right circumflex	1
LAD circumflex	1
Single bypass	43
LAD	37
Diagonal	3
Right	2
Circumflex	1

**Table 4:** Adverse events.

Variable	No. (%)
Mortality	1 (1.7)
Myocardial infarction	1 (1)
Target vessel reintervention	4 (6)
Reoperation for bleeding	2 (3)
Pleural effusion	2 (3)
Pulmonary infection	2 (3)
Post cardiotomy syndrome	1 (1)
